# Hydrogel Polysaccharides of Tamarind and Xanthan to Formulate Hydrodynamically Balanced Matrix Tablets of Famotidine

**DOI:** 10.3390/molecules190913909

**Published:** 2014-09-05

**Authors:** Mahboubeh Razavi, Shaik Nyamathulla, Hamed Karimian, Soheil Zorofchian Moghadamtousi, Mohamed Ibrahim Noordin

**Affiliations:** 1 Department of Pharmacy, Faculty of Medicine, University of Malaya, 50603 Kuala Lumpur, Malaysia; E-Mails: nyamantulla@um.edu.my (S.N.); hamed_karimina61@yahoo.com (H.K.); ibrahimn@um.edu.my (M.I.N.); 2 Biomolecular Research Group, Biochemistry Program, Institute of Biological Sciences, Faculty of Science, University of Malaya, 50603 Kuala Lumpur, Malaysia; E-Mail: soheil.zorofchian@gmail.com; 3 Center for Natural Products and Drug Discovery (CENAR), Department of Chemistry, Faculty of Science, University of Malaya, 50603 Kuala Lumpur, Malaysia

**Keywords:** gastroretentive drug delivery system, floating matrix tablet, sustained release, tamarind seed powder, tamarind kernel powder

## Abstract

The gastroretentive dosage form of famotidine was modified using tamarind seed powders to prolong the gastric retention time. Tamarind seeds were used in two different forms having different swelling and gelling properties: with husk (TSP) or without husk (TKP). TKP (TKP1 to TKP 6) and TSP (TSP1 to TSP 6) series were prepared using tamarind powder:xanthan in the ratios of 5:0, 4:1, 3:2, 2:3, 1:4, 0:5, respectively. The matrix tablets were prepared by the wet granulation method and evaluated for pharmacopoeial requirements. TKP2 was the optimum formulation as it had a short floating lag time (FLT < 30 s) and more than 98.5% drug release in 12 h. The dissolution data were fitted to popular mathematical models to assess the mechanism of drug release, and the optimum formulation showed a predominant first order release and diffusion mechanism. It was concluded that the TKP2 prepared using tamarind kernel powder:xanthan (4:1) was the optimum formulation with shortest floating lag time and more than 90% release in the determined period of time.

## 1. Introduction

Polymers are macromolecules composed of a large number of repeating monomer units. Polymers are used as additives in the manufacturing of dosage forms containing pharmacologically active molecules. They play an important role in different pharmaceutical dosage forms as diluents, binders, disintegrants, gelling agents, thickening agents, emulsifying agents, suspending agents, sustained release agents, stabilizers and coating materials. Polymers affect the stability of the prepared formulation and can influence its physicochemical properties and the drug release [1]. Polymers can be natural or synthetic, and are usually high molecular weight compounds with regularly arranged smaller units. Natural polymers are usually polysaccharides and are widely used as excipients in oral drug delivery dosage forms. These natural materials have advantages over synthetic polymers, since they are chemically inert, nontoxic, less expensive, biodegradable, widely available, have less chance of adverse effects and have better patient acceptance [2]. Natural polymers like gums and mucilages are plant-based hydrocolloids abundant in Nature and common in higher plants. Gums are considered to be pathological products formed following an injury to the plant or as a result of some pathological condition, while mucilages are physiological and normal products of metabolism [3].

The tamarind tree, *Tamarindus indica* Linn. is a large, semi-green and dicotyledonous fruit-bearing tree which belongs to the *Leguminosae* family. It is common in Southeast Asia, India, Pakistan, Bangladesh, Myanmar and Sri Lanka [4]. Tamarind seeds are rich in polysaccharides (~65%–72%), which contain glucose, xylose and galactose units. They are high molecular weight polysaccharides (720–880 kDa) [5], which form viscous solutions in water. Tamarind kernel powder is used as a thickening, stabilizing and gelling agent in the food industry [6]. In view of its characteristics it was selected in the present study along with xanthan.

The oral drug administration route is considered as the most convenient, low cost and the safest route of drug delivery. The effectiveness of oral drug delivery depends on several factors such as gastric emptying rate, gastrointestinal transit time of the dosage form, the rate of drug release from the dosage form and the site of drug absorption [7]. Factors such as short gastric residence time (GRT) and highly variable gastric emptying time of dosage forms can influence the drug bioavailability of pharmaceutical oral dosage forms [8].

Several gastroretentive drug delivery systems (GRDDS) aimed at improving the oral delivery of drugs with an absorption window in the upper part of the gastrointestinal tract, have been designed over the past three decades [9]. The gastroretentive dosage forms can be divided into four main classes: (a) floating systems that cause buoyancy above gastric fluid; (b) expandable systems, which limit emptying through the pyloric sphincter due to expansion; (c) bioadhesive systems that cause bioadhesion to the stomach mucosa and (d) high density systems, retained at the bottom of the stomach [10]. GRDDS improve bioavailability of poorly soluble drugs, increase therapeutic efficacy and patient compliance by reduction of the frequency of the drug intake and by maintaining a constant therapeutic level over a prolonged period of time. Floating drug delivery would be beneficial to drugs that; (a) act locally in the stomach; (b) are primarily absorbed in the stomach; (c) are poorly soluble in alkaline pH; (d) have a narrow absorption window and (e) are not stable in the intestinal or colonic environment [9,11].

Famotidine is an H_2_-receptor antagonist, commonly prescribed for treatment of gastric ulcers, Zollinger-Ellison syndrome, duodenal ulcers and gastro-esophageal reflux disease. Orally administrated famotidine is incompletely absorbed from the gastrointestinal tract [12]. Low bioavailability (40%–45%) and short half-life (2.5–4 h) are the main limitations of the therapeutic effectiveness of famotidine. Due to its short biological half-life, multiple doses are needed to maintain a constant plasma concentration of the drug for a good therapeutic response. However, the increased frequency of drug intake is associated with an increased risk of adverse effects like constipation, fatigue, insomnia, bleeding, depression, nausea, confusion and irregular heart beat [13].

Matrix (monolithic) systems are widely used to sustain the drug release from dosage forms in which the drug is dispersed or dissolved homogenously throughout the polymer. Initially the drug is released from the outer surface of the dosage form, and later from the deeper regions of the dosage form. The drug release from deeper regions can be either by diffusion or by polymer degradation, depending on the polymer and the drug properties. Presently famotidine is dispensed in the market as an over-the-counter medication for treatment and prevention of occasional heartburn associated with excessive gastric acid. Famotidine is useful in promoting the healing of ulcers and in reducing ulcer-induced pain. It has been proven effective in preventing the recurrence of ulcers when given in low doses for prolonged periods of time. Thus the primary objective of the present study was to prepare a sustained release gastro- retentive famotidine dosage form for reducing gastric acid secretion in ulcers as well as for maintenance therapy.

## 2. Results and Discussion

The objective of the present study was to formulate floating gastro-retentive matrix tablets of famotidine with optimal delivery of the drug at the absorption site for a prolonged period of time. Hydrogel polymers in combination with gas generating agents are commonly used in the development of floating matrix tablets [14]. In the present study, Sodium bicarbonate and citric acid were used in the formulations as gas forming agents. The agents induce carbon dioxide generation when they come into contact with the simulated gastric fluid [15]. The generated gas gets entrapped in swelling and rapid gelling hydrocolloids and provides buoyancy in the dosage forms, prolonging the presence of dosage form in the stomach. In floating drug delivery systems, incorporation of an appropriate swelling agent can improve buoyancy and system integrity [16].

### 2.1. Characterization of TKP and TSP

The release rate of the drug depends on physicochemical properties, morphology, size of the particles, stability and compatibility of polymers to other ingredients [17]. Therefore, before formulation, TSP and TKP were analyzed for their physicochemical characteristics with the results shown in Table 1.

**Table 1:** Physico-chemical characters of TKP and TSP (*n* = 3).

Characterization Parameters	TKP	TSP
Bulk Density (g/mL)	0.76 ± 0.09	0.5 ± 0.1
Tap Density (g/mL)	0.84 ± 0.08	0.54 ± 0.12
Carr’s Index (%)	9.52 ± 0.08	7.40 ± 0.91
Hauser Ratio	1.10 ± 0.1	1.0 ± 0.12
True Density (g/mL)	1.42 ± 0.08	1.91 ± 0.09
Moisture Content (%)	3.95 ± 0.1	8.57 ± 0.19
pH	5.26 ± 0.4	5.76 ± 0.3

#### 2.1.1. Determination of Bulk, Tapped and True Densities of TKP and TSP

The powder mixture of all formulations was evaluated for bulk, tapped and true density. As shown in Table 1, TKP had higher tapped and bulk density compared to TSP, TKP had a bulk density of 0.76 g/mL and tapped density of 0.84 g/mL, while TSP showed bulk and tapped densities of 0.50 and 0.54 g/mL, respectively. True densities of TKP and TSP were found to be 1.42 and 1.91 g/mL, respectively. The density variations were probably due to the different methods used to prepare TKP and TSP powder as the TKP was dehusked.

#### 2.1.2. Carr’s Index and Hauser Ratio of TSP and TKP

Carr’s index and Hausner ratio parameter are used to indicate the flowability of powders [18]. TSP and TKP had Carr’s indices of 7.40% and 9.52%, respectively, and Hausner ratios of 1.0 and 1.10, respectively (Table 1). Based on the 2011 British Pharmacopeia’s scale of flowability, TSP and TKP powders can be categorized as powders with excellent flowability.

#### 2.1.3. Moisture Content and pH of TKP and TSP

The stability of powders depends on their level of moisture content. The presence of moisture not only promotes hydrolysis, but also favors microbial growth and subsequent decomposition of the materials [19]. The moisture content data showed that TSP contained 8.57% and TKP contained 3.95% moisture (Table 1). The fact that TSP contained a higher percentage of moisture compared to the de-husked powder of TKP, can be explained by the effect of seed husk on protecting the inner part from absorption of humidity. The pHs of TSP and TKP were found to be 5.76 and 5.26, respectively (Table 1). The slightly acidic property could be due to the presence of uronic acid, glucuronic acid or galacturonic acid, which are commonly found in the polymeric structure of tamarind seeds [20].

#### 2.1.4. Swelling Study of TKP and TSP

The highest Swelling index of TKP was 3720% at pH 7 (distilled water), followed by 3650% at pH 1.2, 1210% at pH 6.8 and 430% at pH 7.4. The highest swelling index of TSP was 910% at pH 7, followed by 760% at pH 7.4, 710% at pH 6.8 and 430% at pH 1.2. The results showed that TKP has better swelling ability than TSP. TKP powder had good swelling properties and greater viscosity than TSP, which may be attributed to differences in the hydration kinetics of the two powders and their structural integrity. 

#### 2.1.5. Viscosity of TKP and TSP

**Figure 1:**
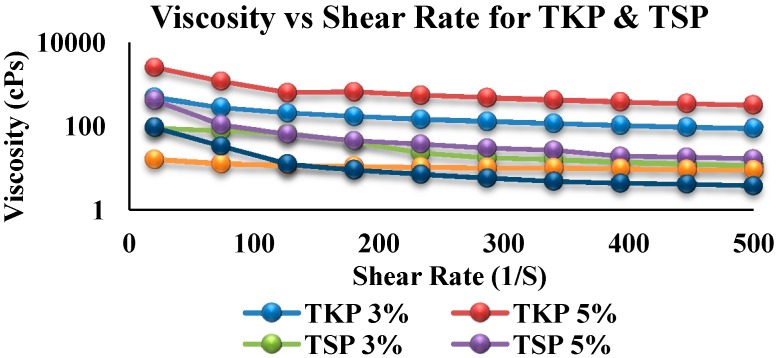
Comparison of TSP and TKP viscosity at different concentrations.

1%, 3% and 5% TKP in distilled water exhibited viscosities of approximately 18, 491.18 and 2564.66 cPs, respectively (Figure 1). Viscosities of 1%, 3% and 5% of TSP in distilled water were approximately 94.08, 93.5 and 418.97 cPs. This result is supported by the swelling ability results. The degree of viscosity of the polymer is an important aspect in formulation development [21]. Substances with high viscosity entrap CO_2_ in the matrix, leading to a density which is less than that of gastric acid (Figure 1).

#### 2.1.6. Differential Scanning Calorimetry (DSC) of TKP and TSP

**Figure 2:**
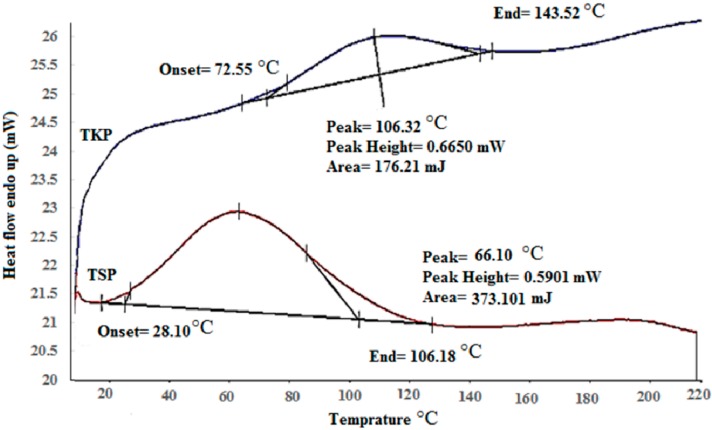
Comparison of differential scanning calorimetry (DSC) curves of TKP and TSP.

The DSC thermograms of pure TKP and TSP showed broad peaks at 108.32 °C and 66.10 °C, respectively (Figure 2), indicating that the melting transition of these powders occurred at these points. The broad melting endotherm peaks of TKP and TSP are an indication of powders with components of multiple thermal characteristics. The difference in melting point of these two powders may be due to changes that occurred during the preparation of TKP and TSP.

#### 2.1.7. Fourier Transform Infrared Spectroscopy (FTIR) of TKP and TSP

**Figure 3:**
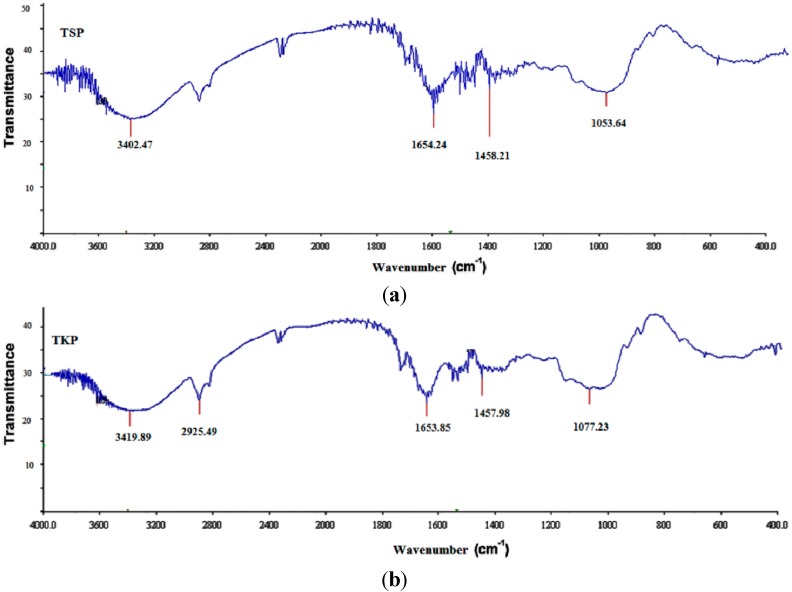
Comparative Fourier Transform infrared spectroscopy (FTIR) of (**a**) TKP and (**b**) TSP.

FTIR spectrophotometry was done to investigate the differences in the molecular structures of TKP and TSP. The spectra for both powders were recorded under the same conditions and are shown in Figure 3. Several major peaks were detected for TKP and TSP. The major peaks of TSP were detected at 1053.64 cm^−1^, 1458.21 cm^−1^, 1654.24 cm^−1^, 3402.47 cm^−1^ and 3717 cm^−1^, while the major peaks of TKP were detected at 1077.23 cm^−1^, 1457.98 cm^−1^, 1653.85 cm^−1^, 3419.89 cm^−1^ and 3712 cm^−1^. The results showed that the spectrum of TKP resembled that of TSP spectrum, indicating that the chemical structure of the powders obtained from husked and de-husked seeds is same.

#### 2.1.8. Scanning Electron Microscopy (SEM) of TSP and TKP

**Figure 4:**
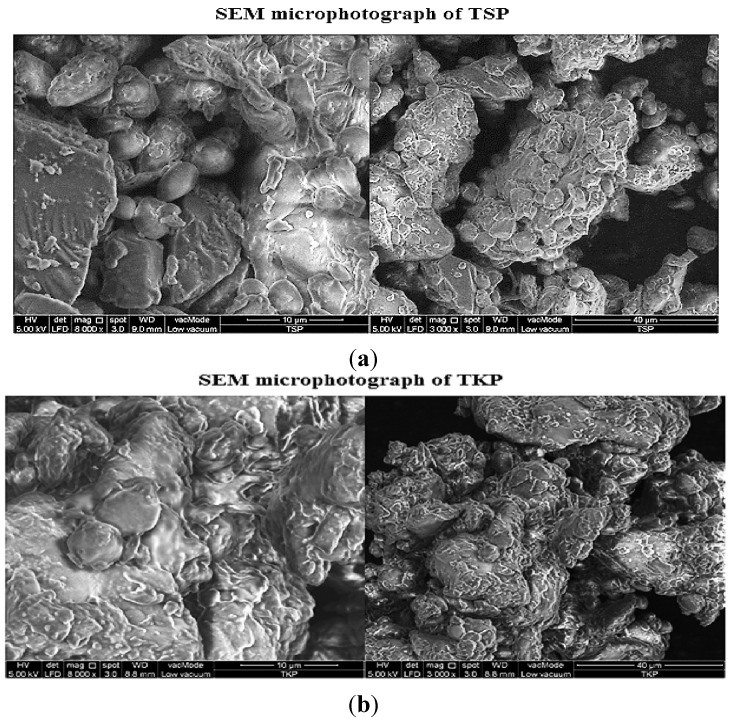
SEM microphotography of (**a**) TSP and (**b**) TKP (Magnification 3000× and 8000×, scale 40 and 10 µm).

The physico-chemical properties and biopharmaceutical behavior of final dosage forms could be affected by the size, and shape of the excipients. The influence of the shape on flow, mixing efficiency, stability, and dissolution and on formulation homogeneity is critical in formulation development so the surface morphologies of TKP and TSP were characterized by SEM microphotographs. Irregular particle sizes with agglomerated shapes were observed in the two powders (Figure 4a,b). The SEM photomicrographs showed that TSP powder has two types of particles, smaller size particles with less surface roughness and rounded edges and large particles with flat, smooth, irregular and sharp edges. The micrograph of TKP reveals that the particles have an irregular shape with smooth but scaly surface morphology. Due to differences in surface morphology of the two powders, it is expected that they may have certain dissimilarities in their behaviors during the tablet preparation and drug release. As expected, TKP powder had good swelling properties and greater viscosity than the TSP one. This may be attributed to differences in the hydration kinetics of the two powders and their structural integrity. Thus, the SEM results of TKP and TSP explained the differences in their morphological appearance and its influence on the polymer properties.

### 2.2. Evaluation of Prepared Matrix Tablets

**Figure 5:**
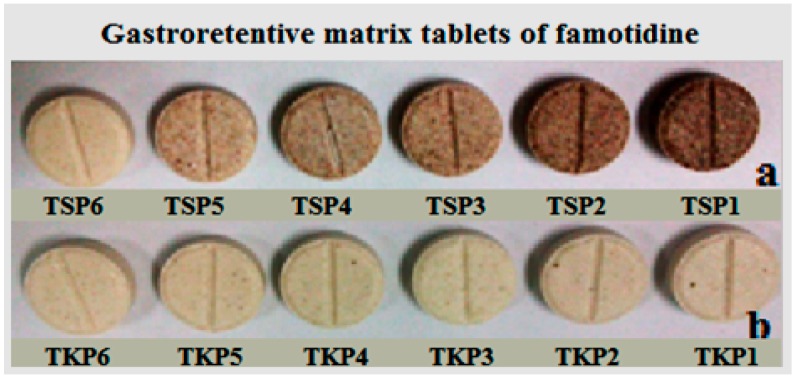
Floating gastroretentive matrix tablets of famotidine, prepared using TSP (**a**) and TKP (**b**) powders.

Floating gastroretentive tablets of famotidine were prepared by a wet granulation technique using xanthan, TKP/TSP, lactose, sodium bicarbonate, and citric acid along with magnesium stearate and talc [22] (Figure 5). TKP 6 and TSP 6 batches had the same tamarind:xanthan ratio and in total 11 prepared tablets were evaluated for characteristics such as average weight, thickness, hardness, friability and drug content. The results of the physical characteristics of the floating tablets are shown in Table 2. All the tablet formulae showed acceptable physicochemical properties and met the USP requirements for weight, drug content, hardness and friability [23]. The thickness of all tablet batches ranged from 2.59 ± 0.00 to 2.61 ± 0.05 mm. The weight of the tablets was found to be in the range of 199.73 ± 1.06 to 201.02 ± 0.89 mg. The drug content uniformity studies revealed that a drug content between 97.16% ± 2.4% and 98.79% ± 0.6% which is acceptable. The hardness values between 81 to 166 N and the percentage friability for all formulae was less than 1%, indicating good mechanical resistance and strength [24].

**Table 2:** Physical evaluation of prepared famotidine matrix tablets.

Formulations	Diameter (mm)	Thickness (mm)	Hardness (N)	Drug Content (%)	Weight Variation (mg)	Friability (%)
TKP1	8.2 ± 0	2.6 ± 0	81 ± 2	98.79 ± 0.6	200.78 ± 1.13	0.391
TKP2	8.2 ± 0.5	2.6 ± 0.5	89 ± 14.5	98.35 ± 0.7	200.18 ± 1.06	0.240
TKP3	8.1 ± 0.05	2.5 ± 0	91 ± 12.6	98.4 ± 0.8	201.52 ± 0.91	0.389
TKP4	8.2 ± 0	2.6 ± 0	123 ± 5	98.38 ± 1	200.76 ± 1.02	0.436
TKP5	8.2 ± 0	2.6 ± 0	154 ± 10.5	97.16 ± 2.4	199.73 ± 1.06	0.436
TSP1	8.2 ± 0.7	2.6 ± 0	118 ± 9	98.69 ± 0.6	200.8 ± 1.12	0.240
TSP2	8.2 ± 0.07	2.6 ± 0.25	136 ± 3.5	98.85 ± 0.7	200.52 ± 0.99	0.29
TSP3	8.1 ± 0.05	2.5 ± 0.9	143 ± 12.6	98.3 ± 0.8	200.74 ± 1.09	0.38
TSP4	8.2 ± 0	2.6 ± 0	148 ± 7	97.98 ± 1	200.68 ± 0.96	0.25
TSP5	8.2 ± 0	2.6 ± 0	156 ± 10.9	98.97 ± 2.4	201.02 ± 0.89	0.21
TSP6/TKP6	8.2 ± 0	2.6 ± 0	163 ± 10.4	98.91 ± 0.4	200.94 ± 1.07	0.48

#### 2.2.1. Floating Lag Time and Total Floating Time of Prepared Matrix Tablets

**Figure 6:**
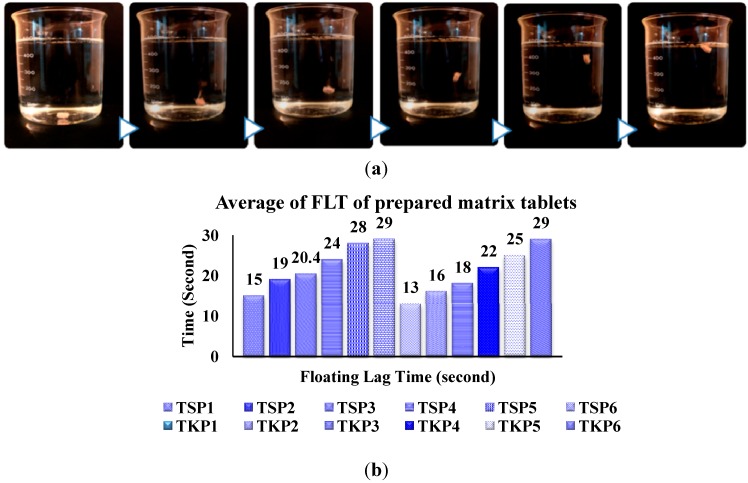
(**a**) Floating of famotidine matrix tablet (TKP2); (**b**) Floating lag time of different formulations of TKP and TSP with equal concentrations of NaHCO_3_.

The floating time (buoyancy) of the tablets depends on two factors: (1) the swelling ability of the hydrocolloid particles on the tablet surface; and (2) the presence of internal voids in the dry centre of the tablet (porosity). These two factors are essential for the tablet to have a bulk density of less than 1 and remain buoyant in the gastric fluid [25]. The *in vitro* buoyancy study results showed quick floating of the tablets within 30 s after placing the tablet in simulated gastric fluid (Figure 6a). Buoyancy mainly depended upon the quantity of sodium bicarbonate and citric acid. Tablets showed good floating ability in the presence of the gas generating agents. From the results, it was concluded that the duration of floating and floating lag time (FLT) decreased with increasing sodium bicarbonate concentration. A concentration of 40 mg of sodium bicarbonate and 20 mg of citric acid was found to be optimal. The floating study showed that tablet’s FLT decreased by increasing TSP concentration and it increased by increasing TKP concentration (Figure 6b). All the prepared formulations exhibited good floating lag times of <30 s and total floating times of more than 24 h.

#### 2.2.2. Differential Scanning Calorimetry (DSC) of the Optimum Formulation (TKP2)

**Figure 7:**
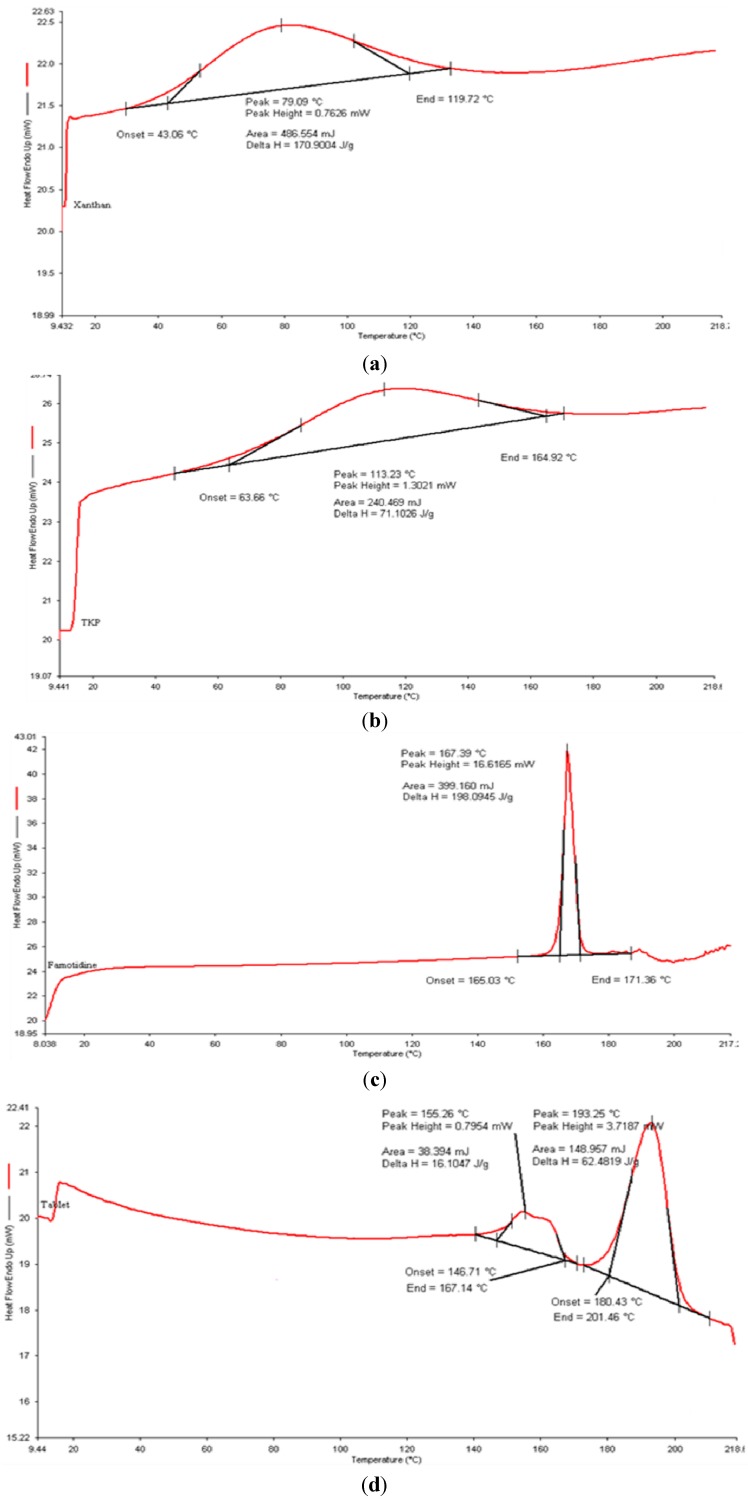
DSC thermogram of (**a**) pure famotidine, (**b**) TKP, (**c**) xanthan and (**d**) crushed optimum matrix tablet of TKP2.

The DSC thermogram of pure famotidine showed a sharp peak as the drug melted at 167.39 °C which indicates that the drug is an anhydrous crystalline molecule of high purity. Meanwhile, a broad peak was observed at 113.23 °C and 70.09 °C from the DSC thermograms of pure TKP and xanthan, respectively, demonstrating that the melting transition of these powders occurred at these points. The melting endotherm of TKP was a broad instead of a sharp peak indicating a polymer with components with multiple thermal character. Peaks corresponding to the melting endotherm of pure famotidine, pure TKP and xanthan were observed on the DSC thermograms of crushed tablets (Figure 7) and there was no significant change in melting point of the drug and powders in crushed tablets and no other appreciable peak was observed. This indicated that there was no interaction between famotidine and the polymers, meaning that the drug and the polymers were compatible [26].

#### 2.2.3. Fourier Transform Infrared (FTIR) Spectroscopy of Optimum Formulation (TKP2)

**Figure 8:**
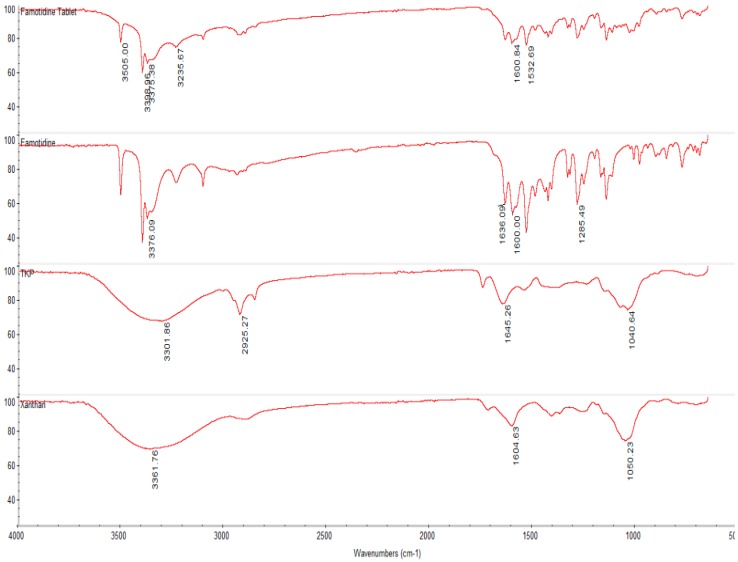
FTIR spectroscopy of pure famotidine, TKP, xanthan and crushed optimum matrix tablet of TKP2.

In the FTIR spectrum, a wavelength range of 950–1200 cm^−1^ is considered the fingerprint region as it allows the identification of major functional groups in polysaccharides such as C–O stretch which peak was obtained at 1040.64 cm^−1^. Other than the fingerprint region, pure TKP and xanthan exhibited a few peaks due to the presence of functional groups such as those obtained at 3301.86 cm^−1^, 2925.27 cm^−1^, 1645.26 cm^−1^, 1040.64 cm^−1^, 3361.76 cm^−1^, 1604.63 cm^−1^ and 1050.23 cm^−1^, respectively. For prepared matrix tablets, peaks were detected at 3505 cm^−1^, 3399.96 cm^−1^, 3375.38 cm^−1^, 3235.67 cm^−1^, 1600.84 cm^−1^ and 1532.68 cm^−1^ and the famotidine peaks were observed at 3376.09 cm^−1^, 1636.09 cm^−1^, 1600 cm^−1^ and 1285.49 cm^−1^. No new peaks were detected in the tablet spectra, indicating that no chemical changes had occurred in the ingredients during tablet preparation [27]. Figure 8 shows the FTIR spectrum of a pure crushed tablet and its ingredients.

#### 2.2.4. X-ray Diffraction Analysis (XRD) of Optimum Formulation (TKP2)

**Figure 9:**
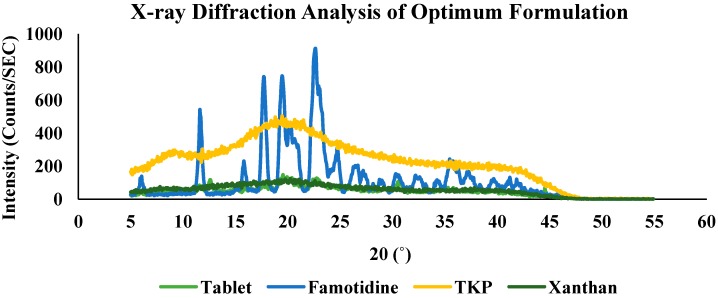
X-ray diffraction of pure famotidine, TKP, xanthan and crushed optimum matrix tablet of TKP2.

X-ray diffraction (XRD) is a rapid analytical technique primarily used for phase identification of a crystalline material and can provide information on unit cell dimensions [28]. The X-ray diffractograms of pure drug, TKP, xanthan and their respective formulation (TKP2) are shown in Figure 9. The X-ray diffractogram of pure drug showed sharp characteristic peaks at 5.15, 11.6, 17.65, 22.55, 25.55, 26.5, 30.55, 32.7, 35.55, 37.6, 39.7 and 41.25 angle (°2θ) indicating the crystallinity of the drug. TKP showed broad peaks at 9.15 and 19.7 and xanthan at 20.4 angles (°2θ) indicating the amorphous nature of both polymers. Xanthan had less characteristic peaks than TKP due to its more amorphous nature. In the TKP2 formulation, there was a decrease in crystallinity of the pure drug as reflected in the diffractogram by a decrease in the intensity of the characteristic peaks. However, there were no major missing peaks in the formulation, except for the peaks of the polymers. Thus, the slight differences observed in the XRD patterns may be attributed to physical interactions as a result of compression during tableting and due to dispersion of polymers while mixing.

### 2.3. In Vitro Drug Release Study

**Figure 10:**
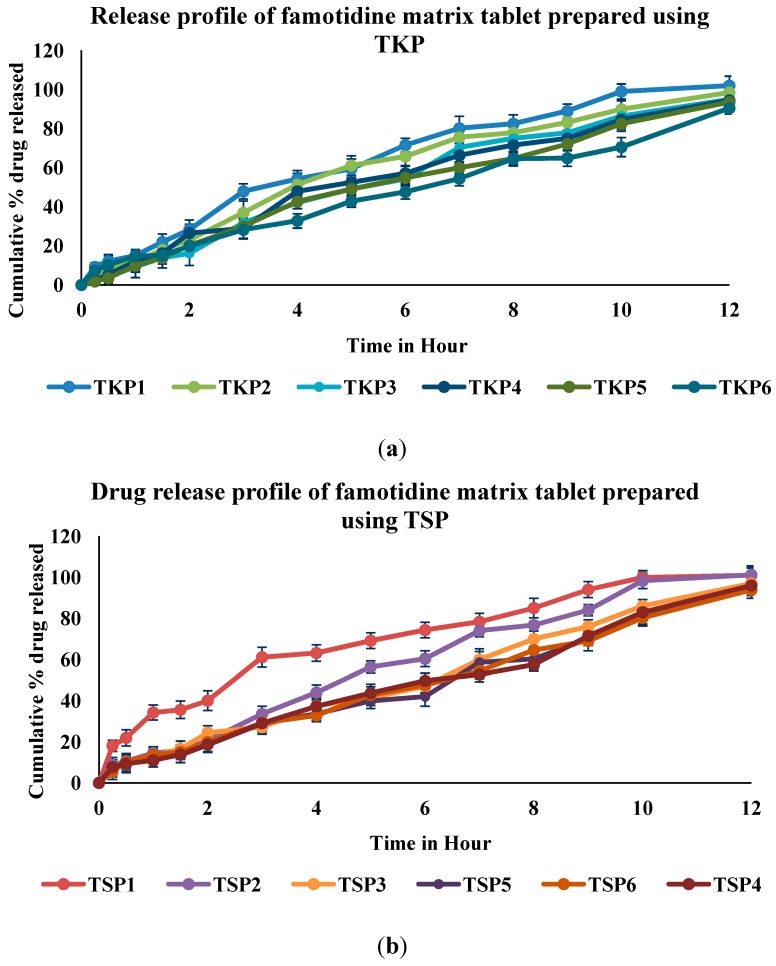
Cumulative percentage of famotidine release from (**a**) TKP and (**b**) TSP formulations within 12 h.

The *in vitro* study of dissolution profiles for all the formulations gave an insight into the effects of polymers on the release profile of the formulations. From the release profiles, it was observed that variation in type of polymers and their concentrations had variable effects on the drug release. These data are illustrated in Figure 10a,b. The effects of xanthan and tamarind seed powder were observed at a constant sodium bicarbonate level. The presence of tamarind seed powder increased the release rate when compared to xanthan. By using the pharmacokinetic parameters of famotidine as reference, the total drug release in 12 h was calculated. To achieve more than 95% release in 12 h, floating tablets should be formulated in such a way as to release the drug in a predetermined and reproducible manner. The release of famotidine from effervescent floating tablets was analyzed by plotting the cumulative percent drug release against time. TKP1, TKP2 and TKP3 contained lesser concentrations of xanthan and their release rates were found to be 102%, 98.5% and 95%, respectively. TKP4, TKP5 and TKP6 with higher concentrations of xanthan, and could release a lesser percentage of drug and their release rates were 94.5%, 93.58% and 90.5%, respectively. The release rates of formulations TSP1‒TSP6 were 101%, 101.2%, 97%, 96%, 94% and 90.5%, respectively.

**Figure 11:**
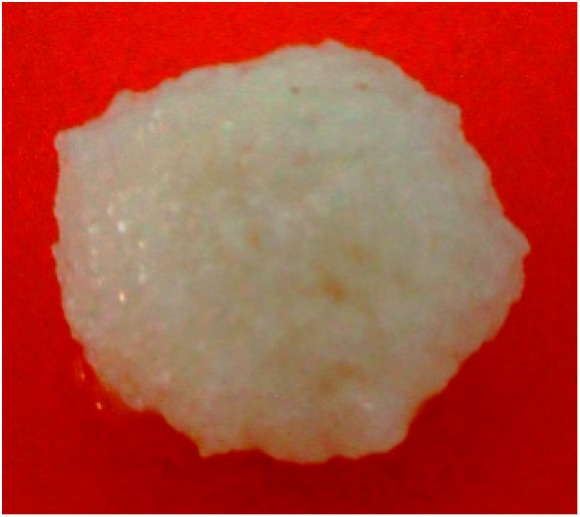
The shape of TKP2 matrix tablet after 12 h of *in vitro* dissolution.

Figure 10a,b shows that TKP1 and TSP1 containing only 50 mg of tamarind powders were able to sustain the drug release for 11 h, but it was noticed that higher concentrations of xanthan in the matrix tablets decreased the drug release. This can be explained by the difference in viscosity and the swelling ability of the two polymers. Xanthan which has a higher molecular weight, forms a gel of a higher viscosity compared to tamarind powders. However, due to its higher molecular weight, the polymer chains are bulkier, leading to less flexibility and hence more time for the polymer and solvent to interact. We observed that floating lag time increased with increasing xanthan concentration. This may be due the high molecular weight of xanthan which increased the FLT. As a result of this, the effective diffusion rate of drug through a matrix containing a higher percentage of tamarind gum is more, leading to higher dissolution rates from these tablets. TKP2 (Figure 11) was chosen as optimum formulation based on its ability to sustain more than 90% drug release for up to 12 h.

The dissolution data of all the formulations were fitted into mathematical models to study their drug release kinetics and mechanisms (Table 3). The mathematical models used were zero order model, first order model, Higuchi release model and Hixson-Crowell model. The regression coefficients of drug release kinetics for all formulations are summarized in Table 3. Based on the “R” values the highest correlation coefficient values were determined and the released data for TKP and TSP formulations seem to fit better with first order and Hixson-Crowell models. This indicates that the drug release of these formulations was concentration dependent and the drug was being released from the surface of matrix instead of diffusing. This was probably due to the strong matrix formed with limited space which result in limited drug release by diffusion.

**Table 3:** Comparison of TSP and TKP regression coefficients of drug release kinetics for Hixson- Crowell, Higuchi kinetic model, first and zero order model.

Regression Coefficients of Drug Release Kinetics				
Formulation	Hixson-Crowell	Higuchi	Zero Order	**First Order**
TKP1	0.9904	0.9864	0.9716	0.9924
TKP2	0.9914	0.9823	0.9757	0.9944
TKP3	0.9934	0.9751	0.9894	0.9904
TKP4	0.9939	0.9843	0.9808	0.9959
TKP5	0.9939	0.9813	0.9843	0.9919
TSP1	0.9884	0.9859	0.9946	0.9787
TSP2	0.9899	0.9757	0.9944	0.9716
TSP3	0.9914	0.9772	0.9929	0.9859
TSP4	0.9787	0.9700	0.9929	0.9591
TSP5	0.9889	0.9705	0.9944	0.9803
TSP6/TKP6	0.9954	0.9813	0.9929	0.9934

## 3. Experimental Section

### 3.1. Materials

Famotidine (white crystalline powder, batch No. 20120201 with 99.61% purity) was purchased from Euro Chemo-Pharma Sdn. Bhd (Selangor, Darul Ehsan, Malaysia). The pure tamarind seeds were kindly provided by Amritum Bio-Botanica Herbs Research Laboratory Pvt. Ltd (Indore, India). Lactose (C_12_H_22_O_11_·H_2_O, M = 360.31 g/mol, conforming to BP, USP, DAB, and Ph.Eur), sodium bicarbonate (NaHCO_3_, M = 84.01 g/mol, conforming to ACS Reag.), microcrystalline cellulose ((C_6_H_10_O_5_)_n_ conforming to BP, USP, DAB, and Ph.Eur), pure xanthan ((C_35_H_49_O_29_)_n_), citric acid (C_6_H_8_O_7_, M = 192.13 g/mol, conforming to BP, USP, DAB, and Ph.Eur), potassium chloride (KCl, M = 74.56 g/mol, conforming to ACS Reag.), hydrochloric acid 37% (HCl, M = 36.46 g/mol, conforming to ACS Reag.), talc and magnesium stearate were purchased from R&M Marketing (Essex, UK). All the materials were used as received.

### 3.2. Preparation of Tamarind Kernel Powder (TKP) and Tamarind Seed Powder (TSP)

**Figure 12:**
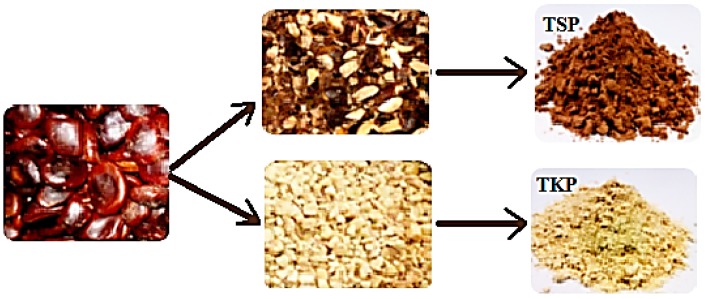
Size reduction of tamarind seeds to TKP and TSP powders.

Tamarind seeds consist of 35% husk and 65% kernel. To make tamarind kernel powder (TKP), strong, dry and clean seeds were de-husked using a sharp knife, the completely dehusked intact seeds and pieces were hand-picked and cleaned to ensure no traces of husk remained adhering to the kernels. A cutter mill (Retsch-Allee, 1-542781 Haan, Germany) was thoroughly cleaned manually by using a brush after dismantling the parts of the mill. Clean kernel pieces of the seeds were size reduced using the above mentioned cutter mill. To make tamarind seed powder (TSP) tamarind seeds (500 g) were fed to the cutter mill and the machine run several times until fine TSP powder was produced, the powder was sieved using a no. 100 sieve to get a particle size 0.149 mm. The TKP and TSP powders were packed in an air tight polypropylene container and stored in a desiccator prior to use. The physical appearance of both TKP and TSP were as shown in Figure 12.

### 3.3. Characterization of TKP and TSP

#### 3.3.1. Determination of Bulk and Tapped Densities of TKP and TSP

For bulk and tapped density, each powder (100 g) was weighed separately into a 250 mL graduated cylinder and the initial volume of powder was recorded as V_0_. The powder was subjected to 300 taps per minute in a tap density tester (Schleunger Pharmatron, Uttigenstrasse, Switzerland). The volume of the powders after tapping was recorded as V_f_. Bulk density and tapped densities of the powders were calculated using the equations below:

(1) $$\text{Bulk}\;\text{Density}=\frac{\text{Weight}\;\text{of}\;\text{powder}\;\left(W\right)}{\text{Initial}\;\text{volume}\;\text{of}\;\text{powder}\;\left(\text{V}0\right)}$$

(2) $$\text{Tapped Density}=\frac{\text{Weight of powder }\left(\text{W}\right)}{\text{Volume of tapped powder }\left(\text{Vf}\right)\;}$$

#### 3.3.2. True Densities of TKP and TSP

A dry 50 mL volumetric flask was weighed accurately, and its weight recorded as W_1_. The volumetric flask was then filled with water up to the 50 mL mark, the surface of the volumetric flask was dried and the flask weighed. The weight was recorded as W_2_. The procedure was repeated using hexane to obtain the weight of the volumetric flask plus hexane (W_3_). About 3 g of powder was transferred into the dried volumetric flask and weighed as W_4_. Hexane was added into the volumetric flask up to the 50 mL mark and the weight determined as W_5_. The density of the hexane used and the true density of powder were calculated by using the equations below:

(3) $$\text{Dnesity of Hexane }\left(\text{Ph}\right)=\frac{\left({\text{w}}_{3}-{\text{w}}_{1}\right)0.9971}{\left({\text{w}}_{2}-{\text{w}}_{1}\right)}$$

where the density of water at 25 °C = 0.9971 g/mL.

(4) $$\text{True Density }\left(\text{ρ}\right)=\frac{\left(\text{W}4-\text{W}1\right)}{\frac{\left(\text{W}3-\text{W}1\right)}{\text{ρh}}-\frac{\left(\text{W}5-\text{W}4\right)}{\text{ρh}}}$$

#### 3.3.3. Carr’s Index and Hausner Ratio of TSP and TKP

Carr’s index and Hausner ratio are the pre-compression parameters that are measures of the relative importance of interparticulate interactions. The flowability of the powders was determined from the results of Carr’s index. The Carr’s index values for TKP and TSP were determined by measuring the initial volume and the tapped volume of known weights of the powders and calculated using the equation below:

(5) $${\text{Carr}}^{\text{'}}\text{s Index}=\frac{\text{Tapped Density}-\text{Bulk Density}}{\text{Tapped Density}}\times 100$$

Hausner ratio was calculated using the equations below:

(6) $$\text{Hausner Ratio}=\frac{\text{Initial Voulme of Powder }(\text{V}0)}{\text{Tapped Volume of Powder }(\text{Vf})}$$

#### 3.3.4. Moisture Content of TKP and TSP

Both polymers were analyzed for the moisture content using a moisture analyzer (HR73 and HG53 Moisture Analyzer, Greifensee, Zürich, Switzerland). Two grams of the sample powders was weighed and placed into the analyzer to get dry and the percent of moisture values.

#### 3.3.5. pH Determination of TKP and TSP

To determine the pH of TKP and TSP, a 0.5% w/v solution of each sample was prepared by adding 50 mg of powders to 100 mL of distilled water. A digital pH meter (Model Seven Easy S20, Mettler-Toledo, Schwerzenbach, Switzerland) was used to read the pH of solutions directly. The pH meter was calibrated by testing the reading of a buffer solutions with a known pH at the room temperature. pH 9.2, 7 and 4 were used for the calibration and then the electrode on an adjustable arm was placed in the sample solution.

#### 3.3.6. Swelling Study of TKP and TSP

Both TKP and TSP were measured for their swelling ability in media of pH 1.2, 6.8, 7.4 and distilled water using a method adopted from Weh, F.H. *et al.* [29], and the swelling index calculated using the formula below:

(7) $$\text{SI}=\frac{\text{Height of the swollen gum}-\text{Initial height of the gum powder}}{\text{Initial height of gum powder}}\times 100$$

#### 3.3.7. Determination of Viscosity of TKP and TSP

Samples of TKP and TSP (1%, 3% and 5%) were prepared with distilled water separately and kept for 24 h to allow the powders to swell. The viscosity was measured using a Brookfield ultra-programmable rheometer (Brookfield Digital Rheometer, Model DV-III, Stoughton, MA, USA) using spindle numbers 42 and 52. The viscosity of the prepared concentration was evaluated with an increment of 5 rpm cone speed till 100 rpm and followed by a decrease of 5 rpm till zero. The results of viscosity changes in response to shear stress and shear rate were determined and compared. Data were analyzed using the Rheocalc software.

#### 3.3.8. Differential Scanning Calorimetry (DSC) of TKP and TSP

The thermal behavior of TKP and TSP were evaluated using a Differential Scanning Calorimeter (Model DSC 6000, Perkin Elmer, Woodland, CA, USA) under nitrogen gas purge of 20 mL/min. A small amount of sample (2.5 mg) was placed into the standard aluminum pan, sealed by crimping and heated over a temperature range of 200–300 °C, at a heating rate of 10 °C/min. The DSC thermograms were analyzed using the Pyris software (PerkinElmer, Inc., Waltham, MA, USA). 

#### 3.3.9. Fourier Transform Infrared (FTIR) spectroscopy of TKP and TSP

A small amount of samples of TKP and TSP were mixed with potassium bromide and made into small, thin discs separately. The discs were analyzed by using an FTIR spectrophotometer (Spectrum RX I FT-IR System, Perkin Elmer). 

#### 3.3.10. Scanning Electron Microscopy (SEM) of TSP and TKP

The surface morphology of TKP and TSP was examined by a field-emission scanning electron microscope (FE-SEM, FEI Quanta FEG 450, FEI Company, Redmond, WA, USA) operated at 5.00 kV at magnifications of 3000× and 8000×, and scales of 40 and 10 µm.

### 3.4. Preparation of Famotidine Matrix Tablets Using TKP and TSP

Prolonged release gastroretentive floating tablets of famotidine were prepared by the wet granulation method [30] using the formulae shown in Table 4. A total of six formulations were prepared for each powder. Four formulations of TKP series and four formulations of TSP series were prepared using TKP and TSP along with xanthan in the weight ratio of 4:1 (TKP2/TSP2), 3:2 (TKP3/TKP3), 2:3 (TKP4/TSP4), 1:4 (TKP5/TSP5), whereas two formulations of TKP and TSP were prepared using only either polymer or xanthan, respectively, in ratios of 1:0 (TKP1/TSP1) and 0:1 (TKP6/TSP6). All ingredients except the lubricants were mixed by the geometrical dilution method. The mixture was then granulated using 1% w/v xanthan as binder solution. The wet granules were passed through a no. 10 sieve and then dried at 45 °C until a constant moisture level was achieved. After drying, the granules were sieved again using a no. 25 sieve to get a uniform granule size. Premixed magnesium stearate and talc which were used as a lubricant were added to the dry granules and mixed well, and the lubricated granules were then compressed into tablets using a single punch manual tablet machine (MTCM-I Manual Tablet Compaction Machine, GlobePharma, North Brunswick, NJ, USA) with a 8 mm round and flat face punch at a compression pressure of 2500 N.

**Table 4:** Formulation composition of famotidine matrix tablets using different concentrations of TKP and TSP.

Ingredients (mg/tablet)	Formulation Codes					
	TKP1/TSP1	TKP2/TSP2	TKP3/TSP3	TKP4/TSP4	TKP5/TSP5	TKP6/TSP6
Famotidine	40	40	40	40	40	40
Polymer *	50	40	30	20	10	0
Xanthan	0	10	20	30	40	50
Lactose	44	44	44	44	44	44
Citric Acid	20	20	20	20	20	20
NaHCO_3_	40	40	40	40	40	40
Mg. Stearate	2	2	2	2	2	2
Talc	4	4	4	4	4	4
Total Weight	200	200	200	200	200	200

*: TKP or TSP powders.

### 3.5. Evaluation of Prepared Tablets

All the prepared formulations were evaluated for hardness, weight variation, friability, floating lag time, total floating time and drug content uniformity.

#### 3.5.1. Weight Variation of Prepared Tablets

Twenty tablets were selected randomly selected and weighed individually using an analytical balance (Model MS-S/MS-L, Mettler-Toledo AG, Laboratory Weighing, Greifensee, Switzerland) and then the average of the 20 tablets was calculated. Individual weights were compared with the average weight and the results recorded.

#### 3.5.2. Thickness and Diameter of Prepared Tablets

Ten tablets were chosen randomly from each formulation of TSP and TKP. Diameter and thickness of these tablets were measured using a hardness tester (Model 6D, Schleunger Pharmatron, Uttigenstrasse, Switzerland). The results were recorded and compared. 

#### 3.5.3. Hardness of Prepared Tablets

Ten tablets were chosen randomly from each formulation. Hardness of these tablets was determined using a hardness tester. The results were recorded and compared.

#### 3.5.4. Friability of Prepared Tablets

All the formulations of prepared tablets were tested for friability using a friabilator (Model Tar10, Erweka, Ensenstam, Germany). Twenty tablets chosen randomly from each batch of tablets were allowed to roll and fall in friabilator at a speed of 25 rpm for 4 min. The tablets were weighed before and after the rotation and the percentage weight loss of tablets was calculated using the formula below:

(8) $$\text{Friability}=\frac{\text{W}1-\text{W}2}{\text{W}1}\times 100$$

#### 3.5.5. Drug Content of Prepared Tablets

Three tablets from each formulation were crushed and a weight equivalent to 100 mg of the drug was weighed, transferred to a volumetric flask and diluted to 100 mL with distilled water. The final solution was filtered and 1 mL of the filtrate was diluted using 9 mL of 0.1 N HCl and absorbance measured at 265 nm using a UV Spectrophotometer (Perkin Elmer Lambda 35 UV Vis Spectrometer).

#### 3.5.6. Floating Lag Time and Total Floating Time of Prepared Tablets

The time taken by tablet to float to the surface of the medium is known as floating lag time (FLT) and the maximum duration time, which the dosage form remains buoyant on the surface of the medium is known as the total floating time (TFT). FLT and TFT were determined by placing a tablet in a beaker containing 900 mL of simulated gastric fluid (pH 1.2) maintained at 37 °C. The time between the placements of the tablet to its float was recorded as the FLT and the overall duration of its float as TFT.

#### 3.5.7. Differential Scanning Calorimetry (DSC) of the Optimum Formulation (TKP2)

Differential scanning Calorimetry analysis was performed with a DSC apparatus (Model DSC 6000, Perkin Elmer,). The small amount of samples (2.5 mg) was placed into the aluminum pan, followed by crimping to seal. The furnace was then heated at a rate of 10 °C/min until 200–300 °C. This was done in a nitrogen atmosphere by purging N_2_ gas at 20 mL/min. The thermograms were analyzed using Pyris software. The test was run for the optimal formulation of TKP2 and its ingredients as active drug, TKP and xanthan.

#### 3.5.8. Fourier Transform Infrared (FTIR) Spectroscopy of Optimum Formulation (TKP2)

The small amount of the TKP and TSP powders were mixed with potassium bromide and made into small, thin pellets separately. The pellets were analyzed by using an FTIR spectrophotometer (Spectrum RX I FT-IR System, Perkin Elmer) this technique is mostly used in organic and inorganic chemistry to determine the functional groups present in molecules.

#### 3.5.9. X-ray Diffraction Analysis (XRD) of Optimum Formulation (TKP2)

The X-ray diffraction spectra of the optimum formulation (TKP2) was analyzed using an XRD diffractometer (Model BTX324, Inxitu, Suite A Mountain View, CA, USA). Dry samples were pressed into pellets and their diffractograms were recorded using Cu-Kα radiation (40 kV, 60 mA). Diffractograms were run at a scanning speed of 2°/min and a chart speed of 2°/2 cm per 2θ.

### 3.6. In Vitro Drug Release Study 

A dissolution test was used to determine the release rate of famotidine from floating tablets (*n* = 3). The test was performed using a United States Pharmacopeia (USP) type II (paddle) apparatus (Copley Scientific Limited, Nottingham, UK), in 900 mL of simulated gastric fluid, at 37 ± 0.5 °C with 50 r.p.m. A 5 mL sample solution was withdrawn from the dissolution apparatus at 0, 0.25, 0.5 1 ,1.30, 2, 4, 6, 8, 10 and 12 h time points and replaced with a 5 mL of fresh simulated gastric fluid (pH 1.2) kept at the same temperature. All the withdrawn samples were diluted using same media and absorbance of sample solutions measured at 265 nm using a UV/Visible double-beam spectrophotometer (Perkin Elmer Lambda 35 UV Vis Spectrometer, Norwalk, CT, USA) [30].

### 3.7. Drug Release Kinetics of Floating Matrix Tablets of Famotidine

The *in vitro* dissolution test data were fitted into mathematical models representing: (i) zero order, (ii) first order, (iii) Higuchi’s and (iv) Hixson-Crowell equations in order to determine the release mechanism and the order of drug release.The cumulative amount of drug released *vs.* time, known as zero order, is used to describe concentration independent drug release rate of the formulation:

C = *k*_o_t (9)

where *k*_o_ is the zero-order rate constant expressed in units of concentration/time and t is the time in hours.

First order, calculated by log cumulative percent drug remaining *vs.* time in hours, describes a concentration dependent drug release from the system:

LogC = LogC_0_− *k*t/2.303 (10)

where C_0_ is the initial concentration of drug and *k* is the first order constant.

Higuchi’s model, as a cumulative percentage of drug released *vs.* square root of time, describes the release of drug based on Fickian diffusion as a square root of time dependent process from a swellable insoluble matrix:

Q = *k*t^1/2^ (11)

where *k* is the constant reflecting the design variables of the system.

Hixson-Crowell cube root law, as the cube root of percentage drug remaining after dissolution test *vs.* time, correlates the release from systems with polymer erosion/dissolution resulting in a change in surface area and diameter of particles or tablets:

Q_0_^1/3^ − Q_t_^1/3^ = *k*_HC_t (12)

where Q_t_ is the amount of drug released in time t, Q_0_ is the initial amount of the drug in the tablets, and *k*_HC_ is the rate constant for the Hixson-Crowell rate equation [31].

## 4. Conclusions 

In this study pulverized tamarind seed powders were successfully characterized and found to have good swelling and tableting character. TKP and TSP could control drug release in the dosage form, and would therefore be suitable for use as swellable polymers in sustained release formulation tablet dosage forms. It was observed that these powders could form a strong matrix tablet when combined with xanthan, and the drug release could be sustained for a longer period of time. We conclude that out of the eleven formulations developed in this study, TKP2 is the optimum formulation as it exhibited a short FLT and more than 90% release in 12 h. TKP2 also showed the effects of tamarind kernel powder in sustaining the drug release for 12 h as this formulation containing only 10 mg of xanthan.
